# From Psychiatric Care to Dermatologic Dilemmas: A Case Report of Risperidone-Induced Bullous Pemphigoid

**DOI:** 10.7759/cureus.69125

**Published:** 2024-09-10

**Authors:** Mohd Rashid Alam, Samant Singh, Astha Agarwal, Manoj Prithviraj, Richa Tripathi

**Affiliations:** 1 Psychiatry, All India Institute of Medical Sciences, Gorakhpur, Gorakhpur, IND

**Keywords:** autoimmune like, drug-induced bullous pemphigoid, infidelity, persistent delusional disorder, risperidone adverse effect

## Abstract

The patient, a 64-year-old male with a history of delusional disorder, first experienced symptoms of suspicion toward his spouse at age 34. He was treated with psychotropic drugs for two years, leading to complete symptom remission and discontinuation of medication. After years of stability, delusions resurfaced two years ago. Amisulpride was ineffective, and risperidone was started but led to itching and plaques on his thighs, which evolved into fluid-filled and hemorrhagic lesions. Diagnosed with bullous pemphigoid (BP) via skin biopsy, he received corticosteroids, dapsone, and antibiotics. Risperidone was discontinued, but delusions reappeared, prompting its reintroduction. New bullous lesions emerged, suggesting risperidone's role in triggering them. Risperidone was replaced with aripiprazole, and melatonin improved sleep. The patient responded well, with both delusional symptoms and skin condition well-managed. This case highlights the need to consider drug-induced BP in elderly patients on antipsychotic medications.

## Introduction

Bullous pemphigoid (BP) is an autoimmune subepidermal blistering disorder, primarily affecting the elderly. It presents as itchy, tense bullae on normal or erythematous skin, commonly on the trunk, limbs, especially the legs, and face. In Western studies, the mean age of onset ranges from 68 to 82 years [1]. However, in India, BP tends to affect a slightly younger population, with a mean age of 60-65 years and a male predominance [2]. Common comorbidities include type 2 diabetes, hypertension, and neurological conditions, such as multiple sclerosis [3]. In addition to neurological conditions, psychiatric disorders also increase the risk of BP. Schizophrenia and psychotic spectrum disorders pose the strongest risk, and as a novel finding, personality disorders have also been shown to precede BP [4]. Other factors, including physical agents, medications, or viral infections, can also trigger its onset through immunogenic or inflammatory mechanisms [5]. A recent case-control study by Lloyd-Lavery et al., which included 86 patients with BP, revealed a notably higher use of loop diuretics among BP patients compared to controls. This association persisted even after accounting for variables such as age, cerebrovascular disease, ischemic heart disease dementia, and hypertension [6]. Similarly, Patsatsi et al. identified angiotensin-converting enzyme inhibitors, anticoagulants, and diuretics as the most frequently used medications among BP patients, with antidiabetics and antiarrhythmics also being noteworthy [7]. However, reports of antipsychotics like risperidone as a potential trigger for BP are exceedingly rare, especially in the Indian context. This case report aims to highlight a rare occurrence of BP potentially induced by risperidone in an elderly patient suffering from persistent delusional disorder, underscoring the importance of monitoring for adverse drug reactions.

## Case presentation

The patient was a 64-year-old male with a long-standing history suggestive of persistent delusional disorder. His symptoms of undue suspiciousness towards his spouse, suspecting her of infidelity, began when he was 34 years old, prompting him to seek psychiatric treatment. He received psychotropic drugs for two years, though the specific medications used during this period are not available. The patient experienced a complete remission of his symptoms, allowing for the discontinuation of his medications. The following years were marked by good mental health and functionality, with no recurrence of his delusional symptoms. The patient had no history of other associated physical comorbidities or was on any chronic treatment.

**Figure 1:**
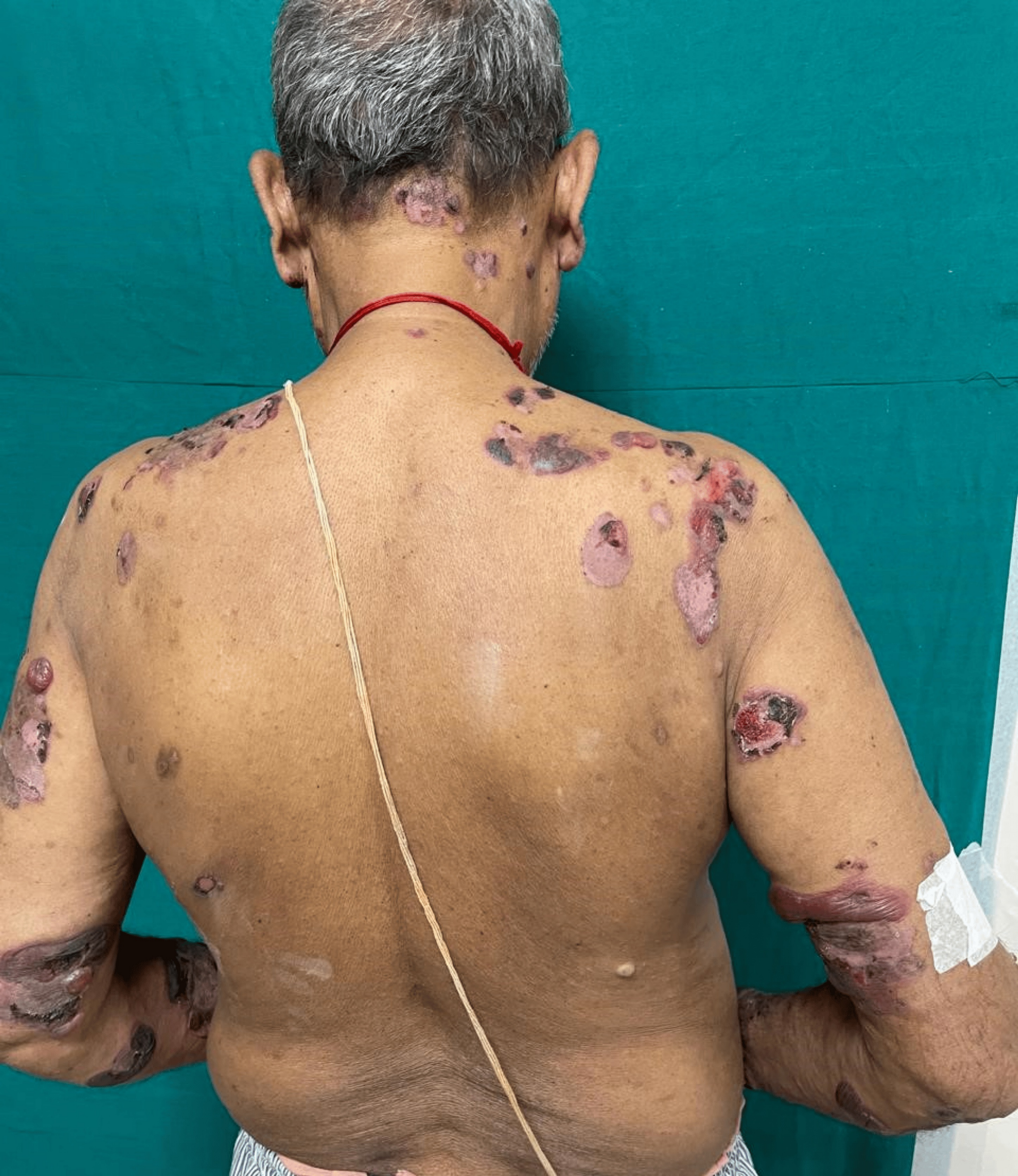
The image depicts various lesions of different sizes across the trunk, shoulders, and arms. Some lesions are fluid-filled, while others have become hemorrhagic. A few have settled, leaving wrinkled skin, and several have healed, showing post-inflammatory hypopigmentation.

**Figure 2:**
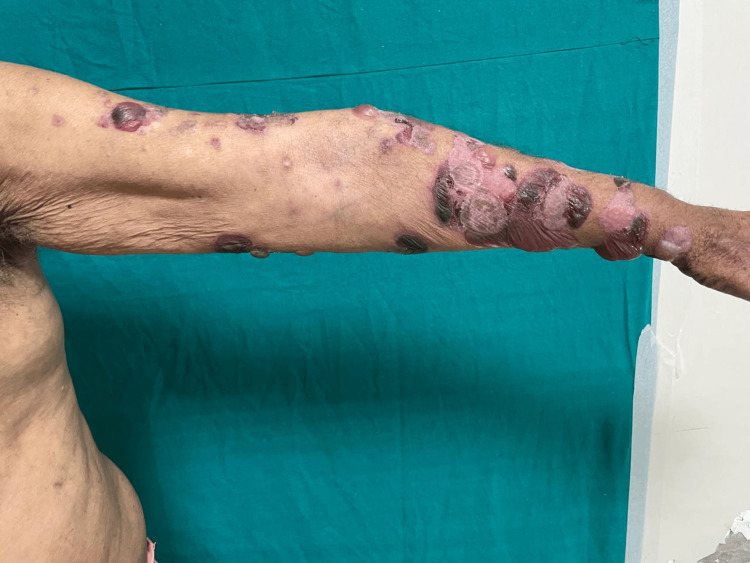
The image shows several lesions clustered together on the forearm. A few are fluid-filled, and some are hemorrhagic, but most have either healed, leaving wrinkled skin, or cleared up with light spots from inflammation.

However, two years before the recent episode, the patient began to experience a resurgence of the delusion of infidelity against his wife. In response, he was given a trial of amisulpride for six months with no significant improvement in symptoms. Risperidone was then initiated and titrated to 6 mg by a private psychiatrist over one month. Within a month of starting the medication, the patient developed itching, and erythematous, edematous plaques over his bilateral thighs. Seeking relief, the patient turned to over-the-counter treatments, which he used for six months with only partial success. Upon discontinuing the over-the-counter medications, approximately 1.5 months ago, the patient noticed the emergence of tense, fluid-filled lesions on his left thigh. Concerned, he sought help from a local practitioner, but within a few days, the lesions spread to his knees, upper chest, back (Figure 1), and bilateral upper limbs (Figure 2). Initially, these lesions were fluid-filled but soon turned hemorrhagic.

**Figure 3:**
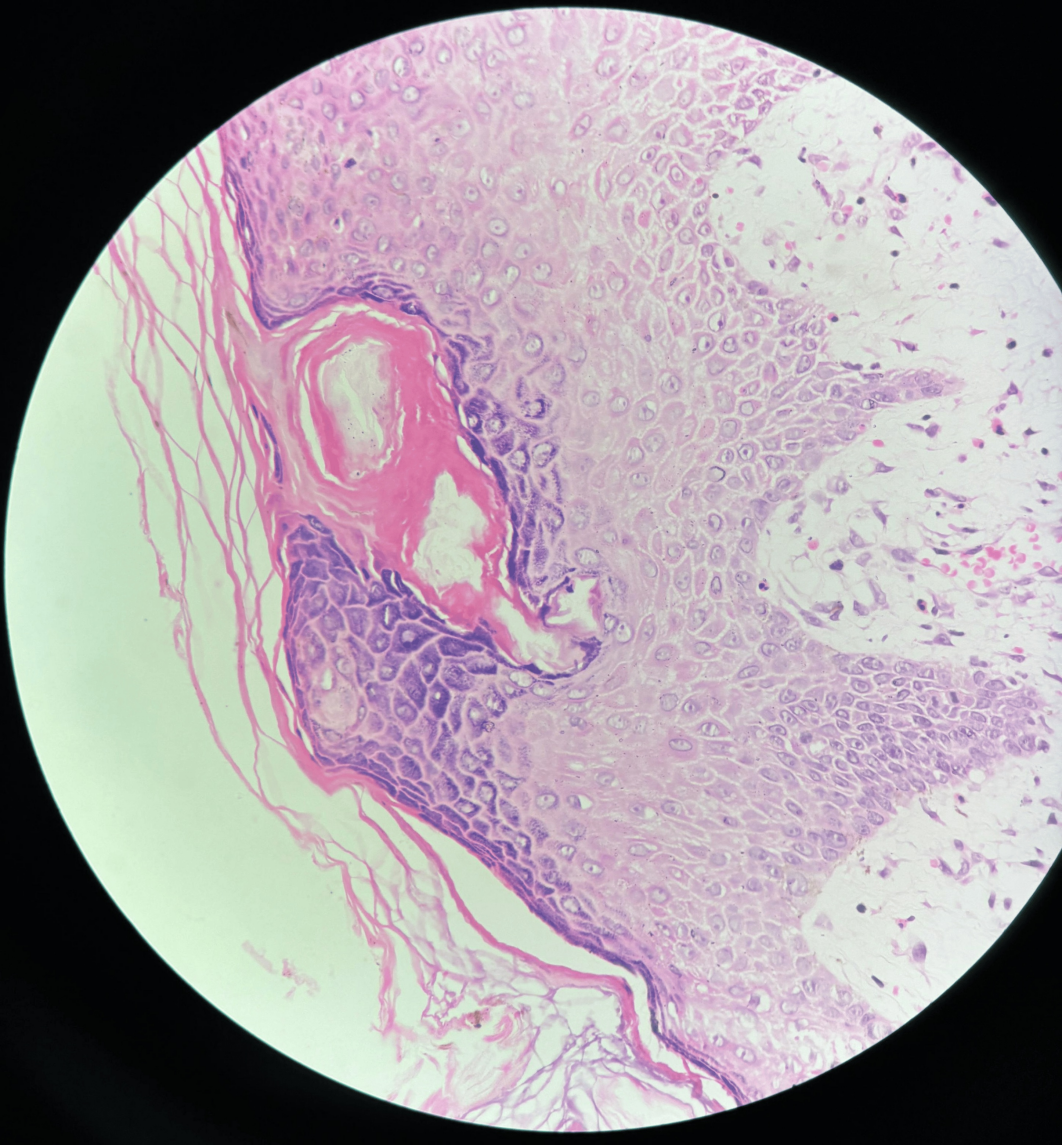
Epidermis showing basket weave orthokeratosis, sub-epidermal bulla present with a cavity containing inflammatory cells (eosinophils)

As the lesions continued to worsen, the patient was referred to a dermatologist, where he was diagnosed with BP, confirmed by a skin biopsy that revealed subepidermal clefts containing inflammatory cells, particularly eosinophils (Figure 3).

Treatment was promptly initiated, including dexamethasone 6 mg OD, mometasone cream to be locally applied on lesion OD, doxycycline 100 mg OD, nicotinamide 200 mg BD, and dapsone 100 mg OD to manage the bullous lesions. Considering the remission of his psychotic symptoms, a psychiatric consultation was sought, and risperidone was tapered and discontinued. Fourteen days later, while the bullous lesions continued to resolve, his suspiciousness toward his wife reappeared, warranting a repeat psychiatric referral. Risperidone was reinitiated, considering the previous response to the same medication. Two days after the reinitiation of risperidone, new bullous lesions started reappearing, raising suspicion that risperidone might be playing a causative role. The antipsychotic was then changed from risperidone to aripiprazole. The patient reported difficulties initiating sleep, so melatonin 3 mg was added to his regimen, improving his sleep quality.

He responded well to the new treatment plan, with his delusional symptoms decreasing and his skin lesions healing, with no new bullous lesions. He was discharged from the hospital within 10 days on prednisolone 15 mg daily, mometasone cream, nicotinamide 250 mg twice daily, dapsone 100 mg daily, aripiprazole 10 mg, and melatonin 3 mg.

During follow-up visits, his delusional symptoms remained under control, and his skin condition stayed stable with no new lesions. This allowed for a gradual reduction of prednisolone, which was tapered down to the current dose of 5 mg per day over three months.

## Discussion

Drugs can trigger BP in people who have a genetic predisposition. Medications may influence the immune system by changing the properties of the epidermal basement membrane or by boosting the immune response [8].

BP develops when autoantibodies target hemidesmosomal components located between the basal cells of the epidermis and the dermis, forming subepidermal blisters. The main autoantigen involved is BP180 (BPAG2 or collagen XVII). Some patients with BP also produce autoantibodies against another hemidesmosomal protein, BP230 (or BPAG1). Current theories suggest that neurological damage or inflammation might expose the BPAG1 neuronal antigen, leading to the production of autoantibodies that mistakenly target the epithelial form of BPAG1 [9]. This mechanism may also explain the association between BP and certain psychiatric conditions, like schizophrenia and persistent delusional disorder, where immune activation is implicated [10].

Medications, such as antipsychotics, can modify the antigenic properties of molecules in the lamina lucida of the basement membrane zone (BMZ), potentially turning them into neoantigens that trigger the formation of anti-BMZ antibodies. This process often involves type I or IV hypersensitivity reactions. Another possibility is that drugs may alter the structure of these molecules, revealing hidden epitopes and provoking an immune response [11].

Although the link between antipsychotic medications and BP is rare, several cases have been reported. risperidone, an atypical antipsychotic commonly used to treat psychiatric conditions, has been occasionally associated with BP. For example, Wijeratne and Webster reported a case of BP in an elderly male with dementia and psychotic symptoms following exposure to risperidone [12]. Similarly, Lozano Pino G et al. described a case of an elderly female who developed BP after treatment with furosemide for acute myocardial infarction and pulmonary edema, along with risperidone for visual hallucinations [13]. In addition, Varpuluoma et al. presented data from a retrospective study showing a small percentage of patients treated with various antipsychotics, with risperidone being the most common [14]. Fabrazzo et al. also reported a case of BP in an elderly male with a history of bipolar affective disorder after administering long-acting risperidone, although the patient had previously taken oral risperidone without adverse skin events [15].

Jedlickova et al. discovered that nearly half (48%) of patients with BP also had a neurological disorder, a rate significantly higher than the 18% observed in a control group with other skin conditions [16]. The skin and nervous system both originate from the neural crest. A recent study showed that antibodies from patients with neurological disease and BP can recognize BPAG1 in both the brain and the epidermis [17]. Considering the temporality between the onset of neurological disease and BP, it is reasonable to suggest that damage to the nervous system during a neurological disorder might expose the neuronal isoform of BPAG1, triggering an immune response that leads to the development of BP [18].

Conversely, while several studies have associated neuroleptic drugs with BP, it remains uncertain whether BP is caused by neurological conditions or the use of neuroleptics, and further research is required to clarify this relationship. This case report emphasizes the need for vigilance in monitoring adverse drug reactions, particularly in patients receiving risperidone, and highlights a rare occurrence of BP potentially induced by risperidone in an elderly patient.

## Conclusions

This case highlights a rare and potentially significant association between risperidone and BP in an elderly patient with persistent delusional disorder. Although BP is known to be linked to various triggers, including medications, the occurrence of BP following the administration of risperidone in this patient underscores the importance of monitoring for drug-induced adverse reactions. The resolution of BP symptoms upon discontinuation of risperidone, followed by their reappearance when the drug was reintroduced, suggests a possible causative role. Clinicians should maintain a high level of awareness when prescribing antipsychotics, particularly in patients with existing psychiatric or neurological conditions, to ensure prompt identification and management of any dermatological complications. Further research is warranted to explore the mechanisms underlying this relationship and to better understand the risks posed by antipsychotic medications in the development of BP.
